# Perioperative Chemotherapy Including Bevacizumab in Potentially Curable Metastatic Colorectal Cancer: Long-Term Follow-Up of the ASSO-LM1 Trial

**DOI:** 10.3390/cancers16050857

**Published:** 2024-02-21

**Authors:** Yawen Dong, Jonas Santol, Birgit Gruenberger, Alfred Lenauer, Friedrich Laengle, Josef Thaler, Gudrun Piringer, Wolfgang Eisterer, Angela Djanani, Judith Stift, Thomas Gruenberger

**Affiliations:** 1 Department of Surgery, Clinic Favoriten, HPB Center, Health Network Vienna, Sigmund Freud Private University Vienna, 1020 Vienna, Austria; 2 Department of Internal Medicine, Hematology and Internal Oncology, Landesklinikum Wiener Neustadt, 2700 Wiener Neustadt, Austria; birgit.gruenberger@wienerneustadt.lknoe.at; 3 Department of Surgery, Landesklinikum Wiener Neustadt, 2700 Wiener Neustadt, Austria; alfred.lenauer@gesundheit-burgenland.at (A.L.); friedrich.laengle@wienerneustadt.lknoe.at (F.L.); 4 Department of Surgery, LHK Oberpullendorf, 7350 Oberpullendorf, Austria; 5 Department of Internal Medicine, Klinikum Wels-Grieskirchen, 4600 Wels, Austria; josef.thaler@klinikum-wegr.at (J.T.); gudrun.piringer@hotmail.com (G.P.); 6 Medical Faculty, Johannes Kepler University Linz, 4040 Linz, Austria; 7 Department of Oncology, Klinikum Klagenfurt, 9020 Klagenfurt, Austria; wolfgang.eisterer@kabeg.at; 8 Department of Internal Medicine, Medical University of Innsbruck, 6020 Innsbruck, Austria; angela.djanani@i-med.ac.at; 9 Department of Pathology, Medical University of Vienna, 2700 Vienna, Austria; judith.stift@meduniwien.ac.at

**Keywords:** potentially resectable colorectal liver metastases, perioperative chemotherapy, anti-VEGF antibody, resectability rate, survival outcomes, wound healing, postoperative bleeding

## Abstract

**Simple Summary:**

The primary aim of this multicenter trial was to evaluate the efficacy, safety and survival outcomes of perioperative chemotherapy with XELOX combined with the anti-VEGF targeted agent bevacizumab in patients with potentially curable metastatic colorectal cancer (mCRC). Six cycles of systemic therapy were administered preoperatively (the sixth cycle did not include bevacizumab) and another six postoperative cycles were given starting 5 weeks after surgery. A total of 35 patients eventually underwent surgery with a resectability (R0) rate of 97%. Three patients developed wound-healing complications, while no postoperative bleeding was reported. Efficacy results for response in 38 eligible patients confirmed an ORR of 66%. Survival analysis revealed a significant improvement in overall survival in the perioperative therapy group when comparing patients who underwent complete perioperative therapy with XELOX and bevacizumab versus those who received XELOX and bevacizumab in the neoadjuvant setting only.

**Abstract:**

In 2007, the ASSO-LM1 trial, a multicenter prospective study, was initiated to investigate the resectability (R0) rate following preoperative combination therapy with XELOX and bevacizumab in patients with potentially resectable colorectal liver metastases. Six cycles of systemic therapy were administered preoperatively, although the sixth cycle did not include bevacizumab, resulting in 5 weeks between the last bevacizumab dose and surgery. Treatment with bevacizumab plus XELOX was restarted for another six cycles postoperatively. In total, 43 patients were enrolled in the ASSO-LM1 trial. Eight patients were ineligible for resection due to protocol violation and progression in two patients. The resectability of operated patients was 97% with 34 R0 resections and one R1 resection. Postoperative morbidity occurred in 22% of patients, of which three operative revisions were related to the primary tumor resection. Efficacy results for response in 38 eligible patients confirmed an ORR of 66%, 31% SD and 3% PD according to RECIST. Preoperative grade 3/4 adverse events were 17% diarrhea, 5% HFS and 5% thromboembolic events. Overall survival significantly differed depending upon the fulfillment of adjuvant treatment in curative resected patients (59.1 mo vs. 30.8 mo). In conclusion, the ASSO-LM1 trial is a hypothesis-generating study confirming the prognostic benefits of perioperative therapy with XELOX and bevacizumab in patients with metastatic colorectal cancer confined to the liver.

## 1. Introduction

Colorectal liver metastases (CRLM) are the most common type of metastases associated with colorectal cancer and occur in around one-third of colorectal cancer patients at the time of diagnosis [1]. Nearly half of the patients will develop metachronous metastatic disease during their lifetime. For resectable liver metastases, surgery is the potentially curative treatment [2]. However, even after R0 resection, around 70% of patients may develop recurrent disease, half recurring in the residual liver [3]. The most plausible explanation for this is the persistence of microscopic residual disease after surgical resection. Therefore, the strategy of combining surgery with perioperative chemotherapy was postulated to potentially eliminate micrometastatic disease and may allow for an improved selection of patients who could profit from surgical resection [4,5]. However, although several randomized clinical trials have been performed investigating the effects and benefits of perioperative chemotherapy in patients with upfront resectable CRLM, the provided results remain controversial and inconclusive with the majority of studies showing no evident improvement of prognosis in this setting [6,7,8,9]. In contrast to that, the use of conversion chemotherapy in patients with initially unresectable CRLM has been widely adopted in the treatment algorithm of CRLM due to the increase in resectability rates; thus, a potential cure may be achieved in this patient cohort [10,11,12].

Constant advances in the development of more effective personalized therapy strategies have not only improved clinical outcomes in patients with mCRC but have also made a wider application of surgery in the treatment of CRLM possible [13]. For instance, the added value of VEGF-targeted monoclonal antibodies such as bevacizumab has long been the subject of much debate [14]. Together with the anti-EGFR antibody cetuximab, it is one of the first approved targeted agents for the treatment of mCRC [15]. In 2004, Hurwitz et al. were among the first authors to demonstrate that by adding bevacizumab to chemotherapy regimens with irinotecan, fluorouracil and leucovorin, patients with mCRC had significantly improved OS, PFS and response rates when compared to those who received chemotherapy alone [16].

Presently, the clinical benefits of the addition of a targeted agent to a cytotoxic doublet or triplet in patients with potentially resectable metastatic disease as induction therapy are well known, and it is considered to be the most effective treatment strategy in mCRC [2]. In brief, the addition of an anti-EGFR monoclonal antibody such as cetuximab to chemotherapy is considered to be the more effective combination in RAS wildtype patients with left-sided primary tumors [17]. Conversely, in patients with right-sided and, in general, RAS mutant metastatic colorectal cancer, the combination of a cytotoxic doublet/triplet and bevacizumab is regarded as the most suitable option provided, as patients may tolerate the intensity of this approach [18].

Back in the 2000s, data on the clinical benefits of perioperative combination therapy with standard chemotherapy and targeted therapy in patients with potentially curable mCRC were limited. For that reason, the ASSO-LM1 trial (NCT00444041) was initiated by the authors in 2007, with the primary objective of determining whether the addition of bevacizumab to chemotherapy with capecitabine and oxaliplatin (XELOX) may further improve the resectability rate in patients with potentially resectable CRLM who did not receive previous treatment for their metastatic disease. Perioperative morbidity, response rates, recurrence-free and overall survival were defined as secondary endpoints. After an extended follow-up time of over 10 years, we now present the long-term survival data, as well as the results of the primary and secondary endpoints, in the following article.

## 2. Materials and Methods

### 2.1. Study Design

The main objective of this study was to evaluate R0 resectability after neoadjuvant systemic therapy with XELOX combined with bevacizumab in patients with potentially curable metastatic colorectal cancer. Further endpoints included the assessment of feasibility with regard to perioperative bleeding and wound-healing impairment, overall response rate (ORR), recurrence-free survival (RFS) and overall survival (OS).

A total of 43 patients were recruited from five different sites (Vienna General Hospital, Hospital Wiener Neustadt, Hospital Barmherzige Brueder Vienna, Hospital Wels and Medical University Innsbruck) in the time from 2007 to 2008. Inclusion criteria were histologically verified colorectal liver metastases, which were treatment-naïve in the metastatic setting and potentially resectable. Baseline evaluation defined if patients were eligible for this trial.

**Figure 1:**
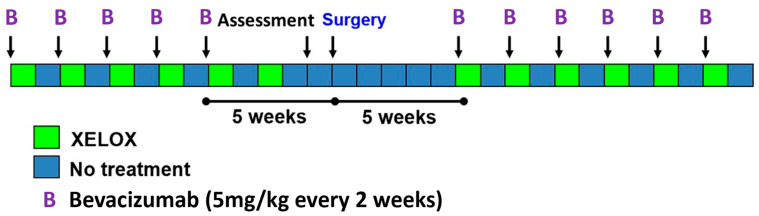
Schematic diagram showing the study design of ASSO-LM1.

**Figure 2:**
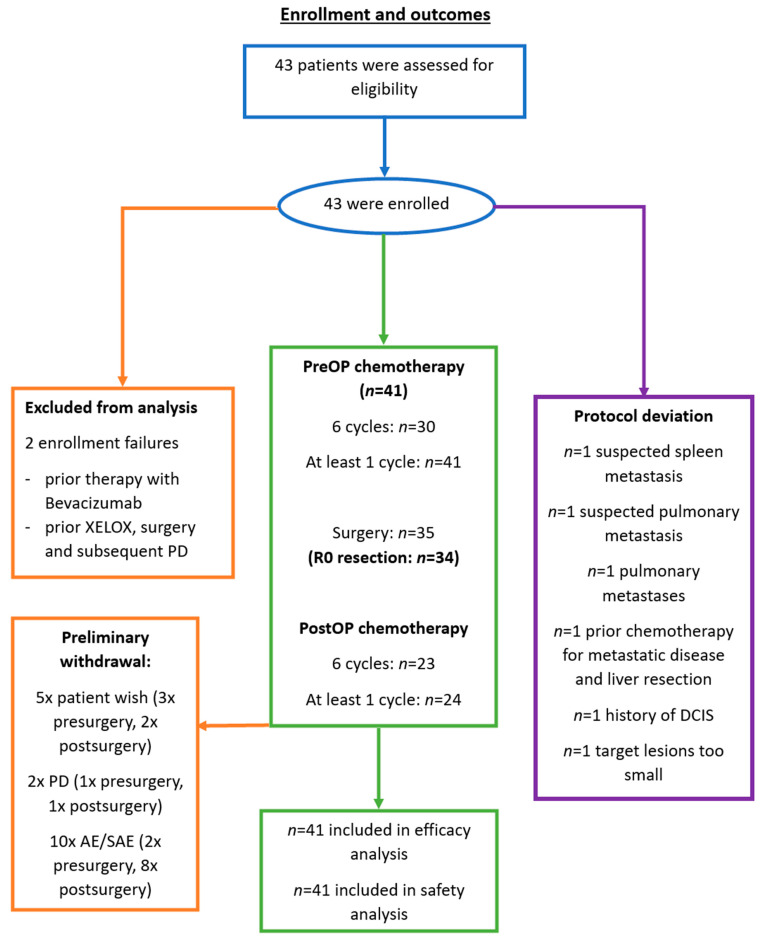
Trial profile. A total of 43 patients were enrolled in the ASSO-LM1 trial. Two patients were excluded from final analysis due to enrollment failures (prior therapy with bevacizumab, prior XELOX therapy followed by surgery and subsequent progressive disease [PD]). From the 41 patients, only 30 patients received the full 6 cycles of systemic therapy in the preoperative setting. Another six patients were excluded from undergoing surgical resection due to several reasons of protocol deviation (see purple square). In total, 35 patients eventually underwent surgical resection. Postoperative systemic therapy was administered to 24 patients in total; of those, 23 patients completed the full 6 cycles of XELOX and bevacizumab. Reasons for preliminary withdrawal included patient wish (3× presurgery, 2× postsurgery), disease progression (1× presurgery, 1× postsurgery) and the occurrence of (serious) adverse events (2× presurgery, 8× postsurgery).

Bevacizumab was administered for five cycles preoperatively at 5 mg/kg once every two weeks. Postoperatively, it was given for six cycles at the same dosage. XELOX was given in parallel for six cycles pre- and postoperatively: liquid oxaliplatin 85 mg/m^2^ q2w and capecitabine 1500 mg/m^2^ BID days 1–7 every two weeks. The sixth preoperative cycle did not include bevacizumab, resulting in 5 weeks between the last bevacizumab dose and surgery (Figure 1). Notably, due to reasons of protocol deviation and preliminary withdrawal, certain patients did not complete the abovementioned treatment scheme (Figure 2).

The efficacy of this treatment combination was evaluated with CT scans according to RECIST criteria prior to the start of treatment, prior to surgery and at the end of the adjuvant therapy and clinical examination. Response rates were assessed by the PIs and after central review, whereas pathological response was assessed centrally according to Rubbia-Brandt. Safety control was closely followed by regular clinical, radiological and laboratory follow-ups. Adverse events were reported according to NCI CTC AE v3.0.

### 2.2. Data Management and Statistical Analysis

Patient characteristics and treatment specifics were obtained from the electronic patient database of the designated study sites. Continuous variables are reported as mean with standard deviation (SD) when normally distributed and as median with interquartile range (IQR) when non-normally distributed. Categorical variables are reported as number of patients with percentages. Fisher’s exact test was performed to compare dichotomous characteristics between groups in order to determine if there are nonrandom associations. The Pearson chi-square test was used for categorical characteristics. Mann–Whitney U test was performed for statistical differences between two groups for continuous characteristics.

As for the secondary endpoints, overall survival (OS) was defined as the time between the date of the first cycle of neoadjuvant therapy and the date of death; otherwise, patients were censored at the time of last contact. Recurrence-free survival (RFS) was defined as the time between the date of the first cycle of neoadjuvant therapy and the date of recurrence diagnosis by imaging. Kaplan–Meier curves were used to demonstrate patients’ OS and RFS. Differences in OS and RFS were compared by log-rank test.

Differences were considered statistically significant when *p* < 0.05. All statistical analyses were performed using SPSS^®^ Version 29.0 (IBM^®^ Corp., Armonk, NY, USA)

## 3. Results

### 3.1. Patient Characteristics

A total of 43 patients were originally enrolled in this multicenter trial. However, two patients had to be excluded from the analysis during the initiation phase due to enrollment failures: one patient had previously received bevacizumab treatment and another patient had been given XELOX therapy palliatively prior to the study and was diagnosed with progressive disease. Therefore, a total of 41 patients with a median age of 66 years were included in the final analysis (Table 1). Of these patients, 68.3% were male, and 78% had synchronous disease. The majority of patients (63.4%) had ECOG 0. Documented comorbidities included the following: 31.7% of patients were smokers, 48.8% had a history of arterial hypertension, 7.3% were diabetic and 4.9% had been previously diagnosed with a gastrointestinal ulcer. Regarding the primary tumor location, 28 patients had a left-sided colon cancer (68.3%) versus 10 patients with a right-sided colon cancer (24.4%); no information was available in 3 patients (7.3%). In 56.1% of patients, the primary tumor resection had been performed prior to trial enrollment. Subsequently, another six patients were considered to be ineligible for resection of the colorectal liver metastases: one patient due to suspected spleen metastasis, two patients who were diagnosed with pulmonary metastases, one patient turned out to have had prior chemotherapy for metastatic disease and liver resection, another patient had been diagnosed with DCIS during the trial, and, in another case, the target lesions were deemed to be too small for surgical resection. As a result, 35 patients remained who underwent surgical resection. With regard to the completion of the pre- and postoperative chemotherapy scheme, preliminary withdrawal from the study was observed due to reasons of patient wish (3× presurgery, 2× postsurgery), progressive disease (1× presurgey, 1× postsugery) and adverse events (2× presurgery, 8× postsurgery) (see trial profile, Figure 2).

### 3.2. Primary Outcome: Resectability Rate

Following preoperative systemic therapy, a total of 35 (85.4%) patients underwent subsequent surgical resection. Of those, 34 (97%) patients received an R0 resection, while one patient had an R1 resection. With regard to the characteristics of resected patients, 8.6% required perioperative blood transfusion, 71.4% were admitted to the intensive care unit for postoperative observation, 77.1% had synchronous metastatic disease, while 51.4% had a lymph node positive primary tumor and a CEA level above the normal range (Table 2). As for the postoperative complications, 17.1% developed a disorder affecting the pulmonary system (pleural effusions, pneumonia), while one patient (2.9%) had a biliary fistula, which was treated conservatively. Three (8.6%) patients had a wound infection, and another three (8.6%) patients had a wound-healing complication. Furthermore, in three (8.6%) patients, revision surgery was necessary, all of which were related to the primary tumor resection and required bowel procedures: in one patient, a protective transversostomy was performed, and abdominal VAC treatment was required due to an anastomotic leak. Another patient also developed an anastomotic dehiscence for which an abdominal revision including lavage and a temporary stoma was necessary. The last of the three patients also received a protective enterostomy for a leaking anastomosis. Of note, one of these patients died of irreversible postoperative liver dysfunction associated with posthepatectomy liver failure (PHLF) following the primary complication of an anastomotic dehiscence.

### 3.3. Secondary Outcomes: Complications, Safety and Response Rate

Efficacy results for response in 38 eligible patients confirm an ORR of 66%, 31% SD and 3% PD according to RECIST. Preoperative grade 3/4 adverse events were 17.1% diarrhea, 4.9% HFS, 2.4% neuropathy and 4.9% thromboembolic events. Postoperative grade 3/4 adverse events were 12.5% neuropathy, 4.2% hyperglycemia, 4.2% leukocytosis and 4.2% diarrhea (Table 3). Histological assessment of resected metastases (*n* = 35) revealed a major pathological response in 56% (with a complete pathological response in 3 patients), a partial pathological response in 28% and no pathological response in 16% of patients. Interestingly, only two cases of sinusoidal obstruction syndrome (SOS) were detected in the 35 resected patients (5.7%).

### 3.4. Survival Analysis: Overall Survival (OS) and Recurrence-Free Survival (RFS)

In the final follow-up phase, survival data were available from 34 of the total 41 patients due to loss of follow-up in 7 patients. When performing the univariate Cox regression analysis, radiological response, surgery and chemotherapy were revealed to be statistically significant (Table 4). With regard to radiological response, the median OS (mOS) in patients with complete response (CR) was not yet reached, whereas patients with progressive disease (PD) had an mOS of 10.0 months, with stable disease (SD) at 30.1 months and with partial response (PR) at 59.1 months. In terms of surgery, resected patients had an mOS of 52.3 months vs. 10.0 months in patients who did not undergo surgery. Similarly, patients with complete perioperative chemotherapy had a significantly longer mOS of 59.1 months vs. 30.8 months in patients who were only given XELOX and bevacizumab in the neoadjuvant setting. Reasons why some patients (*n* = 11) did not undergo adjuvant therapy included patient wishes (*n* = 2), progressive disease (*n* = 1) or adverse events during the neoadjuvant therapy (*n* = 8).

**Figure 3:**
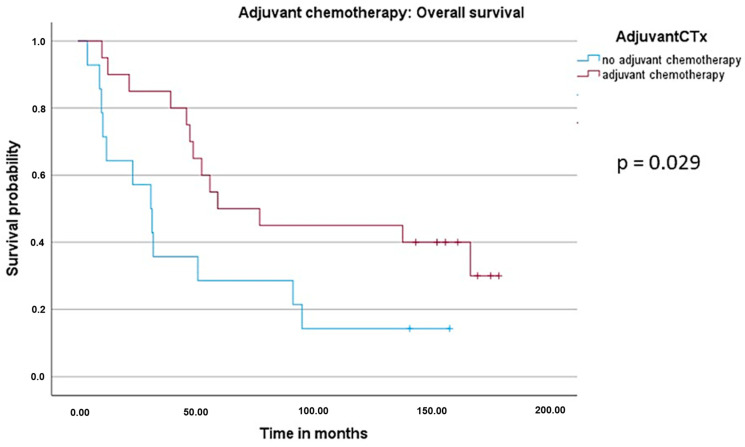
Kaplan–Meier curve and log-rank test for median overall survival (mOS) with regard to perioperative systemic therapy. There was a significant difference in the mOS (*p* = 0.029) between patients who received XELOX and bevacizumab only in the neoadjuvant setting (“no adjuvant chemotherapy”, *n* = 14, 30.8 months) and those who had complete perioperative treatment (“adjuvant chemotherapy”, *n* = 20, 59.1 months).

**Figure 4:**
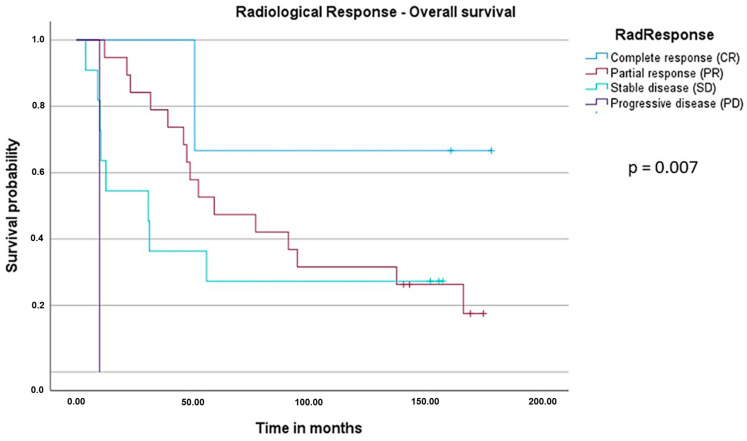
Kaplan–Meier curve and log-rank test for median overall survival (mOS) with regard to radiological response. Radiological response showed statistically significant prognostic value; mOS differed significantly dependent upon the radiological response with a *p*-value of 0.007 (CR not reached, PR 59.1 months, SD 30.8 months, PD 10.0 months).

**Figure 5:**
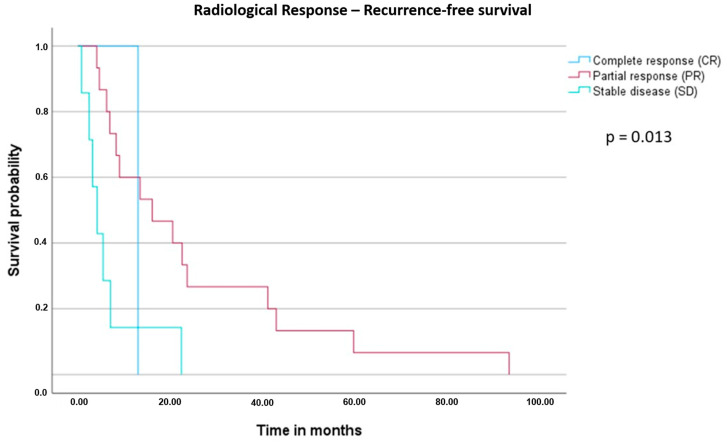
Kaplan–Meier curve and log-rank test for median recurrence-free survival (mRFS) with regard to radiological response. There was a significant difference in mRFS dependent upon the radiological response (*p* = 0.013, CR 13.0 months, PR 16.1 months, SD 4.2 months).

To further evaluate the prognostic value of perioperative XELOX and bevacizumab in patients with potentially resectable liver metastases, the log-rank test was performed (Figure 3 and Figure 4). The parameters “perioperative chemotherapy” and “radiological response” were significant prognostic markers for postoperative outcome. Of note, the radiological response was also associated with a better median recurrence-free survival (mRFS) with 13 months in patients with CR, 16.1 months in PR and 4.2 months in SD (*p* = 0.013, Figure 5).

All in all, due to the limited number of patients, a multivariate analysis was deemed unfeasible in the present study.

## 4. Discussion

The ASSO-LM1 trial aimed to primarily evaluate the resectability (R0) rate in patients with potentially resectable colorectal liver metastases following neoadjuvant treatment with XELOX and bevacizumab, while secondary endpoints included response rates, feasibility, safety and survival outcomes after completion of perioperative systemic therapy. Based on the results from the ASSO-LM1 trial, which only serves for hypothesis-generating purposes due to the small sample size, it may be postulated that perioperative systemic therapy with XELOX and bevacizumab has a tolerable safety profile and adequate feasibility. In the present study, 97% of patients received an R0 resection following neoadjuvant treatment with XELOX and bevacizumab, while postoperative morbidity was at 22% with three operative revisions related to the additional primary tumor resection. Furthermore, our long-term survival analysis showed that overall survival was significantly prolonged in patients receiving neoadjuvant and adjuvant XELOX plus bevacizumab when compared to patients who were not given systemic therapy in the adjuvant setting. Similarly, radiological response was a prognostic marker for a better postoperative outcome, as patients who responded to the neoadjuvant therapy had a significantly improved mRFS and mOS.

When the ASSO-LM1 trial was initiated back in 2007, the data from the EORTC 40983, which were focused on evaluating perioperative chemotherapy with FOLFOX4 for resectable liver metastases, had just been published. Final results revealed a significantly longer 3-year PFS (primary endpoint), but OS did not reach statistical significance since the EORTC 40983 trial was designed to detect an improvement in PFS but was not powered a priori to determine a difference in OS [7]. Nevertheless, the authors concluded that perioperative chemotherapy with FOLFOX4 should be considered as the reference treatment for resectable liver metastases. Consequently, there was increasing interest in studying the potential benefits of the addition of a targeted agent to perioperative chemotherapy in patients with potentially curable metastatic colorectal cancer. The subsequent studies of the EORTC, BOS and BOS-2 did not recruit sufficiently and had to be stopped without any additional data acquired with regard to the addition of EGFR inhibitors or the VEGF inhibitor bevacizumab [19,20]. Cancer Research UK, however, was able to randomize 272 patients with potentially resectable liver metastases into either periOP FOLFOX alone as the standard treatment or FOLFOX plus the EGFR inhibitor cetuximab as the experimental arm with PFS comparison as the primary endpoint. Unfortunately, the trial was closed after an interim analysis fulfilling predefined futility criteria demonstrating a significantly shorter PFS for the chemotherapy plus cetuximab group compared to the chemotherapy alone group [21].

For the ASSO-LM1 trial, the anti-VEGF antibody bevacizumab was chosen for several reasons. Many of the commonly used chemotherapeutic regimens in mCRC have been associated with the development of chemotherapy-associated liver injuries (CALI), which include sinusoidal obstruction syndrome (SOS), steatohepatitis and nodular regenerative hyperplasia (NRH) [22,23,24,25,26]. These disorders can potentially have a negative impact on postoperative outcomes, leading to higher rates of postsurgical morbidities and risks of developing posthepatectomy liver failure (PHLF) [27]. In contrast, the addition of bevacizumab to chemotherapeutic agents such as FOLFOX has been described to be associated with a significant reduction in the rates of developing SOS, NRH and postoperative liver failure in several studies [22,23]. In the ASSO-LM1 trial, only two cases of SOS were detected, while the majority of patients had no signs of SOS in the resected specimen. These multicentric findings support our previous monocentric results providing both a reduction in the incidence of CALI as well as an increase in pathological response with the addition of bevacizumab to FOLFOX [28,29,30]. Interestingly, the addition of the anti-EGFR antibody cetuximab has not been linked with significant changes in CALI rates [22]. Despite its protective effects against CALI, a well-known complication associated with the use of bevacizumab is impaired wound healing and higher risks of bleeding [31]. Therefore, an interval of at least 5 weeks between the last bevacizumab cycle and surgical resection is recommended [2]. In our study, the incidence of wound complications associated with XELOX and bevacizumab was low, and there was no case of postoperative bleeding reported. Therefore, with regard to feasibility and described long-term outcome, it may be suggested that the addition of bevacizumab to perioperative chemotherapy is a safe treatment strategy in patients with potentially resectable mCRC [32].

Neoadjuvant and perioperative chemotherapy is still not accepted and standard of care in many HPB centers, even 15 years after its first positive trial by B. Nordlinger, the EORTC 40983/EPOC study [33]. There were three main intentions to prolong the outcome for patients with potentially resectable colorectal cancer liver metastases: reduce the size of metastases to spare as much liver volume as possible after resection (for potential additional surgeries in case of recurrence), to achieve radiological and pathological response without increasing postOP morbidity induced by CALI and to prolong recurrence-free survival and overall survival compared to surgical resection alone, where we have seen two-thirds of patients recurring within three years of follow-up. ASSO-LM1 has fortunately demonstrated success for all these intentions: responses have been increased compared to FOLFOX/XELOX alone by the addition of bevacizumab from 43% in the EPOC trial to 66% in the current trial; incidence of CALI and postOP complications were sufficiently reduced with the studied regimen, and long-term follow-up showed a significantly prolonged survival in patients who received both neoadjuvant and adjuvant therapy. The value of adjuvant therapy after recovery from surgery is an issue that requires further investigation in the future. Although the long-term overall survival benefit of adding therapy after surgery has not been reported in the recently published CAIRO 5 study [34], these data are eagerly awaited, as up to 64% of patients in this study were not subjected to adjuvant therapy after surgery.

## 5. Conclusions

In conclusion, these data demonstrate that bevacizumab and XELOX provide substantial response rates with controlled adverse events in the potentially curative mCRC setting. In patients eligible for resection after 3 months of neoadjuvant therapy, there was no increase in perioperative morbidity when compared to studies without bevacizumab. Long-term overall survival was significantly improved in our study in patients undergoing postoperative chemotherapy plus bevacizumab. Nevertheless, due to the hypothesis-generating nature of the present study and the small patient sample size, further large-scale trials are warranted in order to confirm the positive effects and to determine the optimal treatment duration of perioperative combination therapy with XELOX and bevacizumab.

## Figures and Tables

**Table 1:** Baseline patient characteristics (*n* = 41).

Characteristics		Descriptive Statistics
Total number of patients		41
Gender	Male	28 (68.3%)
	Female	13 (31.7%)
Age		Median 66 years (38–80 years)
Weight		Median 75 kg (47–120 kg)
Height		Median 170 cm (152–190 cm)
ECOG	0	26 (63.4%)
	1	15 (36.6%)
Medical history	Smoking	13 (31.7%)
	Arterial hypertension	20 (48.8%)
	Diabetes mellitus	3 (7.3%)
	Thromboembolic disease	2 (4.9%)—not active
	Cardiac events	7 (17.1%)
	Gastrointestinal ulcers	2 (4.9%)
	Hematological disease	1 (2.4%)
Primary tumor location	Left-sided	28 (68.3%)
	Right-sided	10 (24.4%)
	Missing	3 (7.3%)
CRLM diagnosis	Synchronous	32 (78.0%)
	Metachronous	9 (22.0%)
Surgery of the primary (prior to study enrollment)		23 (56.1%)
Pathological T stage (*n* = 23)	1	3 (13.04%)
	2	3(13.04%)
	3	14 (60.87%)
	4	3 (13.04%)
Pathological N stage (*n* = 23)	0	8 (34.8%)
	1	11 (47.8%)
	2	3 (13.0%)
	X	1 (4.4%)
Pathological M stage (*n* = 23)	0	7 (30.4%)
	1	15 (65.2%)
	X	1 (4.4%)
Grading of primary tumor (*n* = 23)	1	1 (4.4%)
	2	16 (69.5%)
	3	6 (26.1%)

**Table 2:** Characteristics of resected patients (*n* = 35).

Characteristics		Descriptive Statistics
Blood transfusion		3 (8.6%)
ICU admission		25 (71.4%)
Number of CRLM > 1		19 (54.3%)
Largest CRLM > 5 cm		5 (14.3%)
Synchronous metastases		27 (77.1%)
Lymph node positive primary		18 (51.4%)
CEA above normal range		18 (51.4%)
FONG clinical risk score	0	3 (8.6%)
	1	5 (14.3%)
	2	6 (17.1%)
	3	18 (51.4%)
	4	3 (8.6%)
	5	0 (0%)
Mutational profile	Any KRAS mutation	7 (20.0%)
	KRAS wildtype	17 (48.6%)
	KRAS missing	11 (31.4%)
	BRAF mutation	0 (0%)
	BRAF wildtype	11 (31.4%)
	BRAF missing	24 (68.6%)
Postoperative complications	Pulmonary	6 (17.1%)
	Biliary fistula	1 (2.9%)
	Hepatic failure	1 (2.9%)
	Wound infection	3 (8.6%)
	Wound healing	3 (8.6%)
	Operative revisions (all due to anastomotic leak)	3 (8.6%): ▪ VAC, protective transversostomy (*n* = 1) ▪ Abdominal revision, lavage and protective stoma (*n* = 1) ▪ Protective enterostomy (*n* = 1)
Resection margin	R0	34 (97.1%)
	R1	1 (2.9%)

**Table 3:** Preoperative and postoperative grade 3 and 4 adverse events (AE).

AE Preoperative	Grade 3 and 4 *n* = 41	AE Postoperative	Grade 3 and 4 *n* = 24
Diarrhea	7 (17.1%)	Neuropathy	3 (12.5%)
Hand foot syndrome (HFS)	2 (4.9%)	Hyperglycemia	1 (4.2%)
Neuropathy	1 (2.4%)	Leukocytosis	1 (4.2%)
Thromboembolic events	2 (4.9%)	Diarrhea	1 (4.2%)

**Table 4:** Univariate survival analyses (*n* = 34).

		N	mOS (Months)	HR (CI)	*p* Value
Gender	Male	24	52.3	1.026 (0.424–2.483)	0.954
	Female	10	48.8		
Age	≤66	20	55.8	1.481 (0.672–3.263)	0.330
	>66	14	48.8		
Primary location	Left-sided	25	50.7	0.871 (0.345–2.196)	0.770
	Right-sided	9	55.8		
Syn vs. meta	Synchronous	25	55.8	1.382 (0.575–3.321)	0.469
	Metachronous	9	31.9		
Performance status	ECOG 0	24	52.3	1.336 (0.573–3.118)	0.503
	ECOG 1	10	31.9		
RAS status (missing *n* = 12)	Wildtype	16	50.7	1.774 (0.620–5.082)	0.285
	Mutant	6	21.7		
Fong score (missing *n* = 2)	0–3 (low)	29	55.8	1.584 (0.464–5.402)	0.463
	4–5 (high)	3	48.8		
Radiological response	Complete response (CR)	3	NR	Reference	0.007
	Partial response (PR)	19	59.1	3.178 (0.419–24.127)	
	Stable disease (SD)	11	30.8	4.902 (0.606–39.665)	
	Progressive disease (PD)	1	10.0	56.156 (2.538–1242.455)	
Surgery	Yes	32	52.3	0.179 (0.038–0.851)	0.031
	No	2	10.0		
Chemotherapy (CTx)	Complete periOP CTx	20	59.1	0.418 (0.186–0.938)	0.034
	No adjuvant CTx	14	30.8		

mOS = median overall survival; HR = hazard ratio; CI = confidence interval; NR = not reached.

## Data Availability

The data presented in the study are available upon reasonable request from the corresponding author.
